# Artisanal Gem Mining in Brazil: Evaluation of Oxidative Stress and Genotoxicity Biomarkers

**DOI:** 10.3390/ijerph21070871

**Published:** 2024-07-03

**Authors:** Heberson Teixeira da Silva, Thainá Sprícido Magalhães, Sumaia Araújo Pires, Ana Paula Rufino Santos, Jairo Lisboa Rodrigues, Márcia Cristina da Silva Faria

**Affiliations:** 1 Instituto de Ciência, Engenharia e Tecnologia (ICET), Universidade Federal dos Vales do Jequitinhonha e Mucuri (UFVJM), Teófilo Otoni 39803-371, MG, Brazil; hebersonteixeirasilva@gmail.com (H.T.d.S.); thaina.spricido@ufvjm.edu.br (T.S.M.); sumaiaapires@gmail.com (S.A.P.); anapaularufinosantos@gmail.com (A.P.R.S.); marcia.faria@ufvjm.edu.br (M.C.d.S.F.); 2 Departamento de Análises Clínicas e Toxicológicas, Faculdade de Farmácia, Universidade Federal de Minas Gerais (UFMG), Belo Horizonte 31270-901, MG, Brazil

**Keywords:** mining, occupational exposure biomarkers, oxidative stress, biomonitoring

## Abstract

This study was carried out in the district of Taquaral de Minas, in the municipality of Itinga, located in Jequitinhonha Valley, state of Minas Gerais, which is considered one of the largest yolk-producing regions in Brazil. Miners in gem extraction areas are prone to severe oxidative damage due to their increased exposure to toxic metals, as well as chemical, physical, and biological agents, resulting in diseases such as silicosis. Thus, this work aimed to evaluate occupational exposure in prospectors through biomonitoring techniques using a variety of biomarkers for oxidative stress, genotoxicity, and mutagenicity. Twenty-two miners and seventeen workers who were not occupationally exposed were recruited, totaling thirty-nine participants. The study was approved by the Research Ethics Committee of the Federal University of the Jequitinhonha and Mucuri Valleys. In this study, the levels of total peroxides, catalase activity, and microelements in plasma were evaluated. Additionally, environmental analysis was carried out through the Ames and *Allium cepa* tests. The results of the lipoperoxidation assessment were significant, with increased frequencies in exposed individuals compared to controls (*p* < 0.05), as determined by the Mann–Whitney test. Micronutrients in the blood showed lower concentrations in the group exposed to Fe and Se than in individuals not exposed to these elements. The results of the Ames test and *Allium cepa* test were statistically significant compared to the controls (*p* < 0.05), as determined by the Mann–Whitney test for genotoxicity and cytotoxicity. Thus, the results of the present study indicate possible environmental contamination and a potential risk to the health of miners, which suggests that further studies are important in the region.

## 1. Introduction

Mining is an ancient form of mineral extraction characterized by the high occupational exposure of workers and environmental degradation [1,2,3]. Prospectors in the mining sector are exposed to chemical, physical, and biological agents in the occupational environment, which can lead to the development and aggravation of occupational diseases, respiratory and neurological problems, and poisoning, developed over the course of their labor. Moreover, the environmental impacts of mining can be observed in the contamination of soil, air, and water by toxic metals [3,4].

The health of individuals and ecosystems exposed to toxic agents can be assessed using biomarkers. However, the validation of biological indicators requires particular attention to specificity and sensitivity, and these indicators can be used for various purposes, depending on the purpose of the study [5]. For instance, oxidative stress biomarkers are efficient for biological monitoring purposes and genotoxicity biomarkers, using the test organisms *Allium cepa* and *Salmonella typhimurium*, are suitable for environmental monitoring [6,7].

The occurrence of oxidative stress in the metabolic system is caused by an imbalance between oxidant and antioxidant compounds due to the intense generation of free radicals or to the inadequate speed of their removal from the metabolic system [8,9,10,11,12]. This imbalance is caused by several factors, such as food, chemical exposure, and physical, mechanical and biological agents. This process causes damage to biomolecules, resulting in the loss of their biological actions or even an imbalance in the body’s systems, being expressed through cell and tissue damage [13,14,15,16].

Miners in gem extraction areas in the district of Taquaral de Minas, in the municipality of Itinga, are exposed to toxic metals, as well as chemical, physical, and biological agents, in addition to working in unhealthy conditions [17]. These factors drive the generation of free radicals in these workers’ biological systems and can therefore be toxic. Furthermore, metals have a high accumulation power in living organisms, presenting risks of harm to human health, mainly because some metals, in addition to being easily absorbed by the body, are difficult to remove, accumulating in organs and tissues and causing cell changes, and consequently, mutations, of beneficial or harmful natures [18,19,20,21].

Biomarkers have a number of uses for different purposes. However, they also have specificities, allowing them to be differentiated from each other, thus requiring selectivity in the process of choosing a biomarker for analysis [5]. Thus, the ideal marker will be the one that can express certain pathologies in the community, so it is not necessary to select the best candidates to obtain the necessary information [22].

In addition to verifying the impact of mining activity on human health, assessing its damage to the environment is of interest. One environmental monitoring technique that has been used frequently, with international recognition, is the use of genotoxicity/mutagenicity biomarkers in the test organisms *Allium cepa* and *Salmonella typhimurium* [6,23]. Both tests are effective at evaluating the contamination of the soil by pollutants, as well as in other environments, making it possible to verify the cytotoxic, genotoxic, and mutagenic potential of the substances of interest. In addition, these tests are low-cost, fast, and efficient [6,7,23].

In this context, gem miners in the Jequitinhonha Valley are exposed to chemical, physical, mechanical, biological, ergonomic, and psychosocial factors that may be associated with the high presence of reactive oxygen species in the body [3,17]. Therefore, the quantitative study of biomarkers as a toxicological analysis tool becomes relevant as they can serve as instruments to determine the genesis of occupational diseases in these individuals, enabling effective disease control and prevention for populations facing similar forms of hazardous exposure [1,24].

Therefore, considering the real context of occupationally exposed workers (e.g., to crystalline silica, toxic metals, extreme work environments), this study aimed to investigate oxidative stress parameters (total peroxides in plasma and catalase activity) in plasma as potential early peripheral biomarkers for the monitoring of workers facing occupational exposure, as well as to carry out environmental monitoring (*Allium cepa* test and Ames test) of the studied region using test organisms.

## 2. Materials and Methods

### 2.1. Collection of Samples

**Figure 1:**
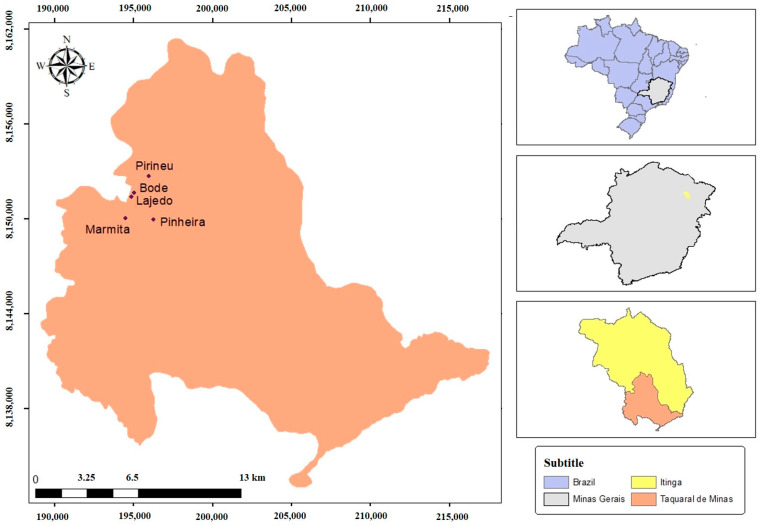
Location of the study area in the municipality of Itinga, district of Taquaral de Minas, Brazil.

In this study, 39 male participants were split into two groups. The group of workers not occupationally exposed comprised 17 men who worked in administrative positions in the city of Teófilo Otoni, Minas Gerais, Brazil, and did not have a history of occupational exposure to crystalline silica and toxic metals. The group of exposed workers consisted of 22 individuals who worked in the mining sector in the Taquaral de Minas district (Figure 1), located in the middle of Jequitinhonha, and who did not have a diagnosis of silicosis. More details about the characteristics of the studied population can be found in the work of Santos et al. (2023) [25].

Exposed workers were recruited by contacting a public establishment in the city, where community health agents helped gather information and invited workers to participate in the study.

The collection, transport, and storage of samples of biological specimens (e.g., blood and urine samples) and soil, soil dust, and stone dust were carried out as described by Santos (2018) [17] and Santos et al. (2023) [25]. Persons with chronic illnesses and those with <1 year of occupational exposure were excluded from the study. All participants completed a questionnaire on health status; lifestyle; drinking, smoking, and exercise habits; diet; medication use; and occupational activities [25].

Soil (SS), soil dust (P), and stone dust (PP) samples from rocks were collected within the five underground mines in the area: Bode (BOD), Pirineu (PIR), Pinheira (PIN), Lajedo (LAJ), and Marmita (MAR). First, a brush with nylon bristles (18 cm long) was used to remove earth dust and stone dust at depths of 0 to 10 cm. Soil sampling was carried out with a manual digging tool at depths of 0 to 20 cm. Three samples from each artisanal mine were collected at specific points: two of the dust samples were collected from the mine soils and one from the rock walls [17,25].

This study was approved by the Research Ethics Committee of the Federal University of the Jequitinhonha and Mucuri Valleys (UFVJM), according to opinion 3.692.758 and CAAE: 22164919.0.0000.5108. All participants were informed about the study and signed an informed consent form.

### 2.2. Oxidative Stress Biomarkers

#### 2.2.1. Total Peroxides in Plasma

Plasma samples (200 μL) were added to 400 μL of thiobarbituric acid 1% *m*/*v* (solubilized in 50 mM NaOH (Sigma Aldrich, St. Louis, MO, USA)), 200 μL of 20% H_3_PO_4_ (Sigma Aldrich, St. Louis, MO, USA), 40 μL of 10 M NaOH (Sigma Aldrich, St. Louis, MO, USA), and 10 μL of 100 mM BTH (butylhydroxytoluene; Pharma Nostra, Rio de Janeiro, Brazil). The solution was homogenized and heated at 85 °C for 20 min. After cooling to room temperature, 1.5 mL of butanol (Neon, Suzano, São Paulo, Brazil) was added. Then, the mixture was vortexed for about 2 min. The upper organic phase was collected, and the concentration was determined from the measurement of absorbance at 535 nm using a UV/visible spectrophotometer (Weblaborsp, São Paulo, Brazil, WUV-M51), as described by Faria (2015) [26]. The calibration curve was prepared with 1,1,3,3-tetramethoxypropan (Sigma Aldrich, St. Louis, MO, USA) at concentrations of 0.2, 0.5, 1.0, 3.0, 7.0, 10.0, 12.5, 15.0, and 20 µM.

#### 2.2.2. Determination of the Antioxidant Enzyme Catalase (CAT) Activity

The CAT activity was determined in plasma samples via absorbance decay, monitored using spectrophotometer readings. The enzymatic assay used to evaluate the catalase activity was performed using H_2_O_2_ ((Merck, Rahway, NJ, USA; 10 mM) as a substrate and a buffered medium, using potassium phosphate as the buffer (Êxodo científica, São Paulo, Brazil; pH 7.0; 50 mmol·L^−1^). As the reaction progressed, decomposition of H_2_O_2_ occurred via the action of the enzyme, and this process was detected by monitoring the decay of the monitored absorbance for 5 min via spectrophotometry at a wavelength of 240 nm, as described by Aebi (1984) [27].

### 2.3. Environmental Assessment through Genotoxicity and Mutagenicity Studies in Soil Samples

The samples were dried at room temperature until complete removal of moisture, and then, subjected to a shaker table procedure with a rotation speed of 115 rpm at 20 °C for 24 h in an aqueous solution at a soil-to-solvent ratio of 1:2 g·mL^−1^, according to Brazilian standards (NBR 10005) (ABNT, 2004). Following this, the solution was centrifuged at 13,000× *g* for 15 min at 4 °C. Then, it was vacuum-filtered with a 0.45 μm membrane to extract toxic compounds from the leached fraction [28].

#### 2.3.1. *Allium cepa* Test

The *Allium cepa* test was conducted as described in previous studies, with some modifications [29,30,31], to analyze the cytotoxicity, genotoxicity, and mutagenicity of soil samples. Seed germination was performed in individual Petri dishes with 3 mL of the extraction solution, previously sterilized in 10% nitric acid, and kept incubated for 5 days at a temperature of 28 ± 0.5 °C in a germination chamber (Fanem 347 CDG, Thermo Scientific, Waltham, MA, USA) containing the extraction solution obtained from soil samples from the regions studied. For the negative control (C−), ultrapure water (18.2 MΩ cm) was used. After 5 days of germination, the roots were measured and transferred to Eppendorf-type tubes containing Carnoy solution (75% ethanol (Sigma Aldrich, St. Louis, MO, USA) and 25% acetic acid (Isofar, São Paulo, Brazil)) for 24 h and stored at 4 °C in a refrigerator. Subsequently, the Carnoy solution was replaced by a 70% ethanol solution and samples were stored until the slides were made. Before slide preparation, the 70% ethanol was removed and the roots were washed 3 times with ultrapure water. Then, the water was replaced with 1 M HCl (Isofar, Rio de Janeiro, Brazil) to carry out acid hydrolysis of the roots for 9 min in a water bath at 60 °C. Soon after, the roots were subjected to Schiff’s reagent (Merck, Rahway, NJ, USA) for two hours to carry out staining in the absence of light. At the time of preparation, the roots were transferred individually to the slide, and their coifs were cut and counterstained with a drop of 2% acetic carmine (Sigma Aldrich, St. Louis, MO, USA) for 8 min, followed by gentle maceration, for cell exposure and assembly using Entellan (Sigma Aldrich, St. Louis, MO, USA). In the analysis, 500 cells per slide were analyzed, totaling 5000 cells per point. The analysis parameters were as follows: chromosomal aberrations, determined by counting losses, breaks, bridges, and c-metaphase, among others; and micronuclei, determined by the frequency of micronuclei and chromosomal breaks. In the microscopic cytotoxicity analysis, mitotic cell divisions were observed for each sample, and the mitotic index (MI) was calculated using Equation (1), whereas for the macroscopic study, the inhibition rate (CTI) was used, calculated according to Equation (2). The total number of cells were scored.

(1) $$MI=\frac{Number\;of\;dividing\;cells}{Total\;number\;of\;cells\;observed}$$

(2) $$CTI=\frac{\left(Average\;root\;length\;of\;(c-)-Average\;root\;length\;of\;the\;sample\right)}{Average\;root\;length\;of(c-)}\times 100$$

#### 2.3.2. Ames Test

The Ames test was performed according to the protocols established by Maron and Ames (1983) [32] and Hott et al. (2021) [30]. The TA98 and TA100 strains were isolated from previously prepared master plates and cultivated for 16 h in nutritive broth at 37 °C with shaking at 170 rpm in a shaker (Thermo Scientific MaxQ 6000, Thermo Fisher Scientific, Waltham, MA, USA). Before the spontaneous reversal test, histidine dependence and the presence of rfa mutations, uvrB deletions, and plasmid PKM101 were tested to ensure that the strains were suitable for the test. For the spontaneous reversal test, which was performed in triplicate, 0.1 M phosphate buffer at pH 7.4 (0.5 mL), bacterial culture (0.1 mL), and the samples under study were added to test tubes with lid screws, which were then incubated at 37 °C for 30 min before adding molten surface agar. The temperature was stabilized at 55 °C, and then, poured onto plates containing minimal agar. The metabolization test proceeded in the same way, only replacing the buffer with the same amount of the S9 mixture (Sigma Aldrich, St. Louis, MO, USA). For the negative controls, ultrapure water was used, and for the positive ones, agents that prove the reversion characteristics of each strain were used. The positive control mutagens used without metabolization for the two Salmonella typhimurium strains were nitroquinoline 4-N-oxide (Sigma-Aldrich, St. Louis, MO, USA) for TA98, at 0.05 mL per plate, and sodium azide (Sigma-Aldrich Chemical Co., St. Louis, MO, USA) at a concentration of 0.1 mL per plate for TA100. For the activation test, aminoanthracene (Sigma-Aldrich, St. Louis, MO, USA) was used as a positive control for both strains. The test strains used were obtained from MOLTOX and were stored at 4 °C until use. Then, the number of reverse colonies was counted. Statistical analysis was performed, and the mutagenicity ratio (MR) was calculated using Equation (3).

(3) $$MR=\frac{Number\;of\;Reverserson\;Test\;Board}{Number\;of\;Reverserson\;Control\;Board}$$

A compound is considered mutagenic if it produces a reproducible increase in the number of reversible colonies that is higher than twice the number of spontaneous reversers, MR ≥  2. The analyses were performed in triplicate, and the statistical data were recorded using the Graph Pad Prism 8.0.2 software. The Mann–Whitney test was used to evaluate the significant difference between groups.

### 2.4. Statistical Analysis

In the static analysis, the Mann–Whitney test was applied to the test results (total peroxyls in plasma, total catalase activity, micronutrient levels, *Allium cepa* bioassay, and the Ames test) for comparison between the exposed and control groups. ANOVA tests were used to verify whether there were statistical differences according to the age of the individuals concerning the concentrations of total peroxides in plasma and total catalase activity, as well as to analyze the relationship between the studied mines for the samples analyzed in the *Allium cepa* bioassay. Descriptive statistics (mean and standard deviation and coefficient of determination (R^2^)) were used to evaluate the numerical results. An association was considered statistically significant when the *p*-value was less than 0.05. The software used to process and analyze statistical data were GraphPad Prism (GraphPad, version 8.0.2, La Jolla, CA, USA) and Microsoft Excel^®^ (Microsoft Office 365, Washington, DC, USA).

## 3. Results and Discussion

### 3.1. Total Peroxides in Plasma

**Figure 2:**
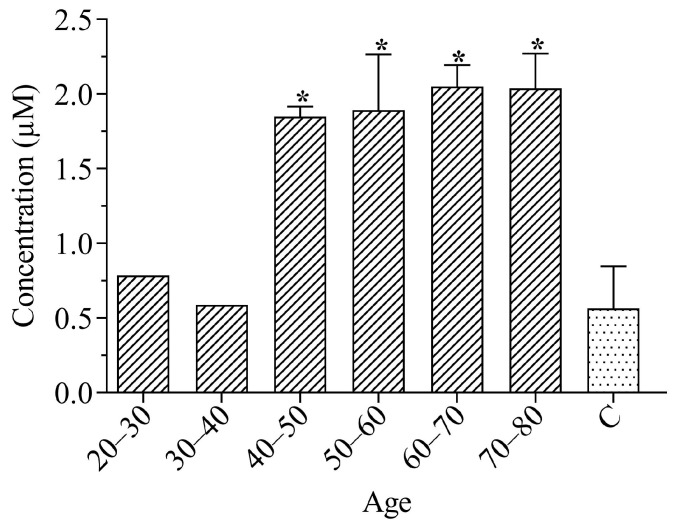
Representation of average values of total peroxides in miners from the Taquaral district and in participants from the control group. Asterisks (*) indicate significant differences (*p* < 0.05) for samples when compared to the control (C).

The results of the analysis of the total peroxides in the plasma of the study participants are shown in Figure 2, using an aqueous calibration curve with a value of r^2^ = 0.999:

It can be seen in Figure 2 that the total peroxide results were statistically significant (*p* < 0.05), using the Mann–Whitney test, when compared to the control for individuals over 40 years old. The ANOVA test showed a significant difference (*p* < 0.05) between the study groups. Thus, through multiple comparisons, it was noted that individuals aged 20–30 and 30–40, when compared with those aged 40–50, 50–60, 60–70 and 70–80, showed statistically significant differences.

The values for peroxidation found may be linked to the lifestyle and dietary factors of individuals [9,33]. Santos et al. (2023) [25] reports in his study that workers are exposed to unhealthy working conditions in environments with high concentrations of metals and silica during the gem extraction process, and most workers in this sector are also smokers and consume alcohol. Such aspects are related to the lipoperoxidation of biomolecules [9,34]. In this context, Demirci-Cekic et al. (2022) [8] and Yang et al. (2022) [10] assert that alcohol and its toxic metabolites increase reactive oxygen/nitrogen species (ROS/RNS), causing damage to vital organs. Additionally, the greatest oxidative damage was observed in the serum and erythrocyte membranes of patients with alcohol-related disorders, correlating with high levels of malondialdehyde (MDA). This reinforces the hypothesis that lifestyle factors, including alcohol and smoking, may exacerbate the detrimental effects of oxidative stress on workers exposed to unhealthy environmental conditions.

Another factor that corroborates the obtained results is occupational exposure to crystalline silica due to the drilling, extraction, and processing of minerals and the production and handling of materials containing silicon dioxide [35]. Contact between these particles and the lung parenchyma causes a constant local inflammatory reaction and oxidative damage, which can lead to an increase in the level of MDA, cell death, and DNA damage [1,36,37]. Furthermore, studies show that exposure to free radicals generated by crystalline silica damages biological membranes, triggering the process of lipid peroxidation, both in vitro and in vivo [1,37,38,39].

In accordance with the results obtained in this study, Nardi et al. (2018) [40] and Peruzzi et al. (2019) [1] demonstrate increased values for MDA in miners and in individuals exposed to silica. Similarly, significant increases in MDA levels were noted for gold miners over 40 years old when compared to the group that was not exposed [24,40]. Thus, the results presented in Figure 2 may indicate a possible exposure of this group of workers, as well as the potential development of future diseases in this group, as previous studies have reported a relationship between higher plasma levels of total peroxides and cardiovascular, liver, and neurodegenerative diseases, cancer, and diabetes [41,42,43].

### 3.2. Total Catalase Activity

**Figure 3:**
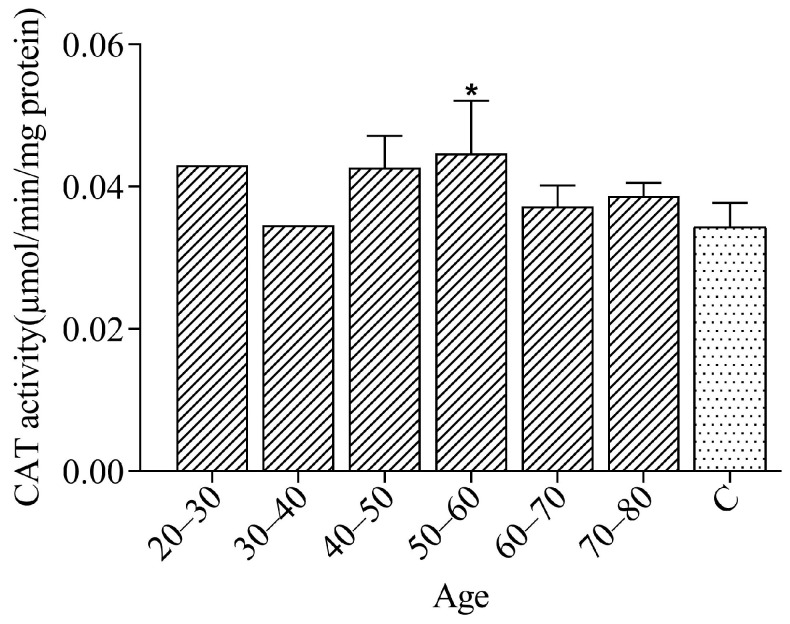
Quantification of mean values of enzyme catalase activity in miners from the Taquaral district and in the control group. Asterisks (*) indicate significant differences for (*p* < 0.05) samples when compared to the control (C).

Catalase is an antioxidant enzyme involved in the neutralization of endoperoxides [44]. Its activity in the plasma of miners is high compared to the control group, as shown in Figure 3, but this difference was not statistically significant in all groups according to age.

CAT enzyme levels were increased in mining workers, which may be associated with their chronic exposure to xenobiotics, as well as due to the occupational conditions related to mineral extraction. In this sense, catalase is involved in the decomposition of endoperoxides from the normal cell metabolism, protecting the organism against radical species which would otherwise exert degrading and toxic effects on some cell types, such as erythrocytes [45,46,47].

The statistical analysis (*p* < 0.05) of the results shows that there was a significant difference in the activity of CAT when compared to the control group only in workers belonging to the 50–60 age group. Considering that this enzyme is an antioxidant produced by organisms in response to the excessive production of free radicals and that the occupational exposure of individuals is usually acute, CAT activity in other groups may be a reflection that the organism has not yet adapted to such conditions [48].

Catalase activity, although not statistically significant for some groups, is consistent with chronic exposure to contaminants, as well as occupational exposure, which will consequently lead to greater production of hydroperoxides in the cells. Many cells in the immune system can produce ROS, including alveolar macrophages, which play a fundamental role in the body’s defense against dust particles [40,49,50].

The microsomal metabolism of endogenous and xenobiotic compounds, including environmental pollutants, also has a role in the process of ROS formation. During the process of the phagocytosis of inhaled particles, superoxide radicals (O_2_^•−^) are generated, and their dismutation results in hydrogen peroxide (H_2_O_2_). In the presence of transition metal ions, such as ferrous ions or cuprous ions, H_2_O_2_ is converted to the potent oxidizing hydroxyl radical (OH^•^) through the Fenton reaction. Thus, there is evidence that the inhalation of toxic occupational substances and environmental pollutants leads to excessive in vitro production and in vivo generation of OH^•^ radicals during phagocytosis [8,40,51,52].

### 3.3. Micronutrients

Micronutrients are necessary in trace amounts in the body and play essential roles in numerous biochemical and physiological processes. They are exogenous sources of antioxidants that protect DNA and other biomolecules from oxidative damage. Minerals such as selenium, zinc, copper, iron, and magnesium are considered antioxidants, as they each participate in some way in processes mitigating the effects of oxidative stress, act against the formation and neutralization of free radicals, and favor the repair and reconstitution of damaged biological structures [8,53]. Furthermore, the body’s antioxidant system has several antioxidant mechanisms that depend on the action of these minerals to prevent and combat the damage caused by oxidative stress [54]. Table 1 presents the values of these elements in the studied population as reported by Santos (2018) [17] and Santos et al. (2023) [25] and determined using inductively coupled plasma mass spectrometry (ICP-MS).

One hypothesis to explain the change in micronutrients in the study group compared to the unexposed controls would be the difference in the consumption of the main sources of exogenous antioxidants. Thus, the decrease in the availability of these minerals affects metalloenzymes, which are important components of antioxidant defense. For example, it is known that superoxide dismutase is dependent on copper, zinc, and manganese, as well as selenium catalase [8,10].

The reduction in these minerals that participate in the antioxidant defense mechanism may have left the lungs of these individuals more susceptible to oxidative attack, resulting in an inflammatory response and respiratory diseases, such as silicosis. The lungs of miners are constantly exposed to reactive oxygen species or free radicals through the inhalation of crystalline silica. Therefore, there is an imbalance between the production and elimination of radical species, exacerbating oxidative stress and leading to consequent apoptosis and production of local chemotactic molecules, increasing vascular permeability and the inflammatory process [55,56].

The individuals from the 30–40, 40–50, 50–60, 60–70 and 70–80 age groups showed lower mean serum levels of minerals when compared to the control group. Therefore, it is noteworthy that one of the main functions of Se is its antioxidant potential, acting as a cofactor of glutathione and glutathione peroxidase in conjunction with catalase [57]. So, the levels of this enzyme decrease in Se-deficient organisms, and Se supplementation restores its normal activity [58]. In addition, Se strengthens the immune system, exerting preventive effects against diseases such as cancer, atherosclerosis, arthritis, stroke, cirrhosis, and emphysema, in addition to combating poisoning from toxic metals [53,59].

Although not statistically significant, the zinc value in the miner group is below the mean level. Studies report that the deficiency of this element may be associated with a diet deficiency or due to excessive alcohol consumption [60,61]. Zinc acts in the strengthening of cell membranes and protects the liver against possible damage caused by free radicals; therefore, its absence can cause a series of neurological disorders [62,63].

For the copper value, there is a lower mean value for the exposed miners in the 30–40 and 50–60 age groups when compared to the control group; for the other age groups, there is a small decrease, but without statistical significance. This may be associated with the reduction in Zn [60]. This micronutrient is essential for many enzymes that help break down or build tissues in the body; in addition, it is necessary in the production of RNA, protection against the oxidation of polyunsaturated fatty acids and in helping to maintain healthy cell membranes. A lack of copper in the body weakens the immune system [60].

The mean value of Fe showed statistical differences for the 50–60 and 70–80 age groups. This micronutrient is an essential constituent of several proteins, including enzymes, cytochromes, myoglobin, and hemoglobin. Thus, some studies suggest that deficiency in this mineral predisposes individuals to infections [64]. However, in the presence of ions of this element, products from lipid peroxidation can be rapidly amplified, potentiating cell damage [65].

### 3.4. Evaluation of Cytotoxicity, Genotoxicity and Mutagenicity by the Allium cepa Bioassay

#### 3.4.1. Cytotoxicity Analysis

The cytotoxicity of soil samples (soil (SS), soil dust (P), stone dust (PP)) was studied using the mitotic index (MI), inhibition rate (CTI), and microscopic and macroscopic parameters, characterized by the total number of cells in mitosis. This metric was used because root growth is driven by the frequency of cell division in the tissue, and the decrease or increase in the number or rate of dividing cells can be correlated with the toxic effect of a substance [66].

**Figure 4:**
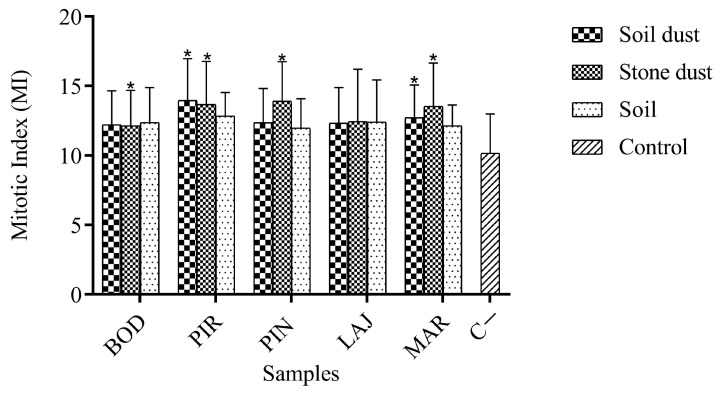
Results of mitotic index values, expressed as mean ± standard deviation. Asterisks (*) indicate significant differences (*p* < 0.05) for samples when compared to the negative control (C−). Abbreviations: BOD = Bode; PIR = Pirineu; PIN = Pinheira; LAJ = Lajedo, MAR = Marmita.

Table 2 presents some parameters used in the cytotoxicity study. The values for the mitotic indices show that there are statistically significant differences (*p* < 0.05) between the negative controls and the PIR P, PIR PP, PIN PP, MAR P, and MAR PP, samples according to the Mann–Whitney test. The other samples in the study showed mean values for the MIs that were numerically greater than the negative controls (Figure 4). MIs statistically higher than the negative controls may indicate alterations in the growth and development of the exposed organism resulting from chemical exposure. The consequences of this exposure are increased cell division, which can be harmful to cells, leading to disordered proliferation and even the formation of tumor tissues [67].

**Figure 5:**
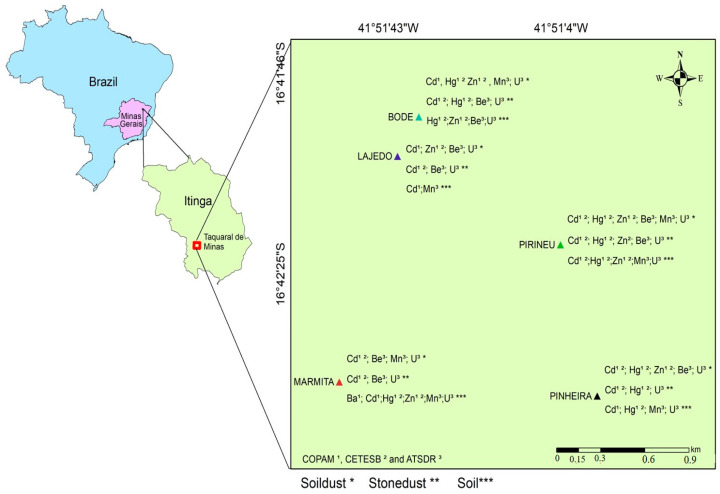
Toxic metals above the levels allowed by national (COPAM—State Council for Environmental Policy and CETESB—Environmental Company of the State of São Paulo) and international (ATSDR—Agency for Toxic Substances and Disease Registry) laws found in the mines studied by Santos (2018) [17] and Santos et al. (2023) [25].

As described in the study carried out by Santos et al. (2023) [25] and presented in summary form in Figure 5, the regions studied presented high values of metals in the soil, exceeding limitations set by current national and international legislation [17,25]. This fact may be related to the statistical differences observed for the MI, as the presence of metals in high concentrations in the soil can cause an increase in the MI and reflect the cytotoxicity that directly affects growth and root elongation. A decline in this parameter compared to the negative control can cause cell death and other lethal effects in the body [23].

Corroborating the results obtained in the cytotoxicity study, Leme and Marin-Morales (2009) [67] describe the relationship between metal concentrations and MI, where a higher metal concentration tends to indicate a higher cytotoxicity in the sample. Thus, the change in the MI of *Allium cepa* meristem cells suggests that the presence of high concentrations of heavy metals in the soil caused cytotoxic effects in the test organism under investigation. In this same context, Carruyo et al. (2008) [68] and Chandra et al. (2005) [23] report that due to the presence of toxic metals, changes in root growth and mitotic index were observed.

**Figure 6:**
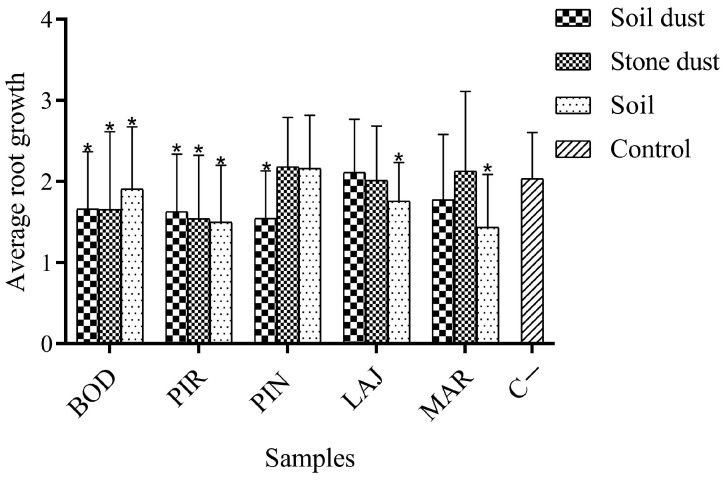
Results of mean root growth values after exposure to aqueous extract of mining soil samples from the Taquaral district of Minas Gerais, Brazil, expressed as mean ± standard deviation. Asterisks (*) indicate significant differences (*p* < 0.05) for samples when compared to the negative control (C−). Abbreviations: BOD = Bode; PIR = Pirineu; PIN = Pinheira; LAJ = Lajedo; MAR = Marmita.

Figure 6 presents the mean values for root growth. Bonciu et al. (2018) [6] state that this indicator is considered the most important macroscopic parameter in the *Allium cepa* test, as data on germination provide information on the lethality of the analyzed substance. Furthermore, root growth is associated with MI and is the main cytotoxicity parameter in *Allium cepa*. The BOD P, BOD PP, BOD SS, PIR P, PIR PP, PIR SS, PIN P, LAJ SS, and MAR SS samples showed statistically significant differences (*p* < 0.05) when compared to the negative controls using the Mann–Whitney test. These results may be correlated with the high concentration of metals found in the region’s soil, since, as described by Cunha Neto et al. (2003) [69], the presence of toxic metals can inhibit root growth in this bioassay.

#### 3.4.2. Analysis of Genotoxicity and Mutagenicity

To study the genotoxicity and mutagenicity, chromosomal aberrations and micronuclei were observed, respectively. Chromosomal aberrations are characterized by structural changes at different stages of cell division that can occur spontaneously or as a result of exposure to contaminants. On the other hand, an increased number of micronuclei may indicate the existence of a mutagenic substance, being the result of alterations to chromosomes and the mitotic process resulting from acentric fragments or entire chromosomes that were not incorporated into the main nucleus during the cell division cycle.

**Figure 7:**
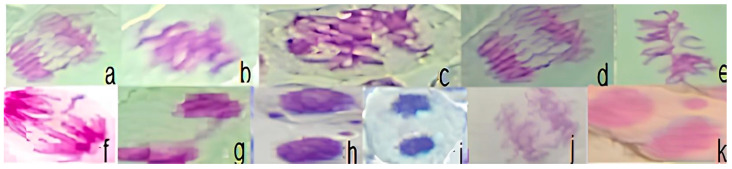
Photomicrographs of chromosomal and micronuclei alterations observed in the *Allium cepa* test. (**a**,**j**) = bridging; (**b**,**e**) = loss; (**c**) = c-metaphase; (**d,f**) = bridging and breaking; (**g**,**i**) = sticking; and (**h**,**k**) = micronuclei.

**Figure 8:**
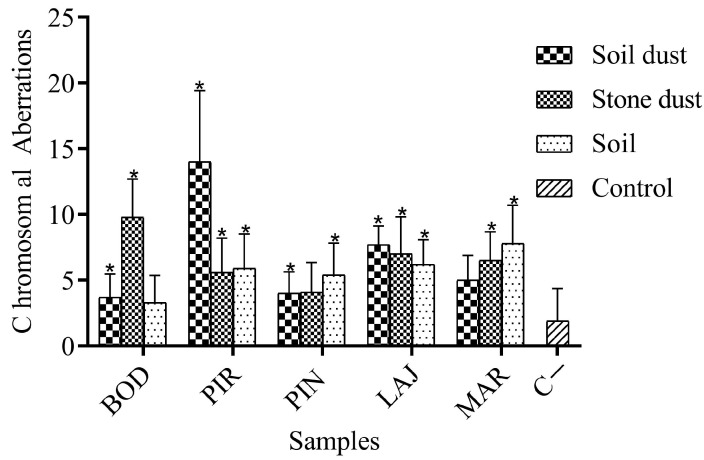
Results obtained for the study of chromosomal aberrations in 5000 meristematic cells of *Allium cepa* after exposure to aqueous extract of soil samples from Garimpos in the district of Taquaral de Minas, Brazil, expressed as mean ± standard deviation. Asterisks (*) indicate significant differences (*p* < 0.05) for samples when compared to the negative control (C−). Abbreviations: BOD = Bode; PIR = Pirineu; PIN = Pinheira; LAJ = Lajedo; MAR = Marmita.

Quantitative analyses of the various chromosomal aberrations (Figure 7) are summarized in Table 3 and Figure 8, with the following abnormalities found in the study: bridging, breakage, cormosonic loss, sticking, c-metaphase, and micronuclei.

Statistical analysis revealed that the frequencies of chromosomal aberrations for the BOD P, BOD PP, PIR P, PIR PP, PIR SS, PIN P, PIN PP, PIN SS, LAJ P, LAJ PP, LAJ SS, MAR PP, and MAR SS samples differed significantly (*p* < 0.05) when compared to the negative controls, as determined using the Mann–Whitney test in the genotoxicity analysis (Table 3 and Figure 8). On the other hand, there was no statistical change (*p* < 0.05) in the mutagenicity analysis (Table 3 and Figure 7), although it is noted that, numerically, the micronuclei values are very high compared to the positive control.

Chromosomal aberrations in the *Allium cepa* test are used to predict the mechanism of action of xenobiotics, such as toxic metals, in biological systems, recognized as important consequences of the actions of these chemical agents in living organisms. High frequencies of chromosomal aberrations have been correlated with a significant risk of developing cancer. Furthermore, such cellular anomalies can cause problems in the reproductive processes of organisms, such as an increase in germ cells and genetic diseases that can be transmitted to later generations [6,66,69,70].

The significant induction of chromosomal aberrations, including the formation of micronuclei, indicates the genotoxic and mutagenic potential of the soil samples under study. Tabassum and Pandey (2021) [71] state that the presence of metals can induce chromosomal breaks, fragments, and micronuclei formation in plant and mammalian test systems. From this perspective, Santos et al. (2023) [25] observed high concentrations of some metals present in the region’s soil, justifying the presence of such anomalies.

In line with the results obtained, Wierzbicka (1999) [72], Achary et al. (2008) [73], and Datta et al. (2018) [74] report in their studies that the presence of toxic metals is associated with genetic alterations. There is also a connection between the abnormalities found and the contaminant present in the sample. For instance, the presence of toxic metals (e.g., Al, Cr, Pb, Cu, Mg, Co, Zi, Mn, Ni, Cd, Hg) is related to c-metaphase, chromosomal bridges, giant cells, and viscosity, occurring mainly in soils with higher metal concentrations. Importantly, there are two ways in which toxic metals can induce genotoxicity: one is cross-linking with DNA and/or protein and the other is the generation of reactive oxygen species [6,23,75].

Toxic substances, such as metals, contaminating the environment may not show acute effects on exposed organisms but may cause reduced survival [76,77], tissue damage, genetic damage to somatic and germ cells, the accumulation of persistent contaminants, and the occurrence of neoplasms. Changes in the cell division index and genetic material can be harmful to an organism and induce severe and irreversible consequences to human or animal health [78,79].

The formation of micronuclei is usually the result of chromosomal breaks/fragments or an anomalous disjunction of chromosomes in the anaphase stage of the cell cycle. Therefore, the presence of micronuclei can be used to assess the effect of clastogenic and aneugenic parameters, revealing the mutagenic action that may or may not be associated with neoplastic transformations. A high value of this anomaly indicates the aneugenic effect resulting from a chromosomal loss and, when reduced, may indicate a clastogenic action resulting from a break in the chromosome. Thus, micronuclei are related to genetic anomalies that can reveal genetic syndromes and autosomal trisomies in humans [6,75].

Although the values for micronuclei (Table 3) do not present statistical significance, it is numerically verified that they are increased compared to the negative controls. Thus, chronic exposure over the span of years can cause damage to human health, in addition to chronic environmental effects. Moreover, interactions between metals or other elements, even at low levels, can have synergistic or additive actions [76].

Table 4 presents the results of the ANOVA/multiple comparisons test carried out between the mines. It is possible to verify that, for the analyzed parameters, only the chromosomal aberrations and the mean growth of the roots showed a statistically significant difference (*p* < 0.05) when comparing the samples, as shown in Table 4. Such changes may be due to regional variation in the soil composition, leading to the distinct values of certain elements. Furthermore, there may be changes in the concentrations of elements along their horizons.

### 3.5. Ames Test

**Figure 9:**
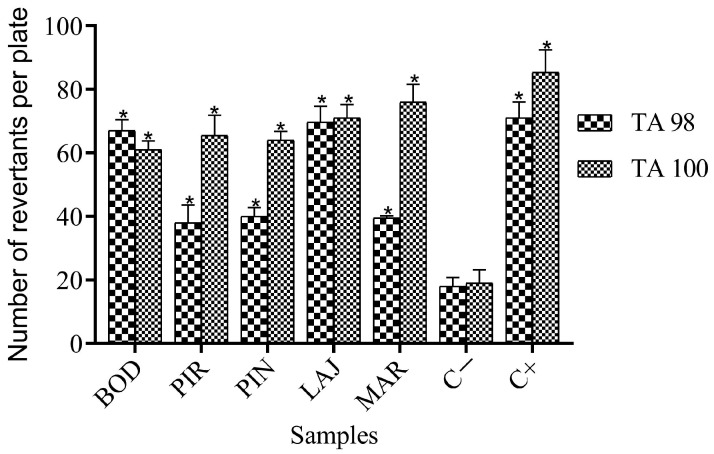
Results obtained for the Ames test without metabolization when testing aqueous extract of soil samples from mines in the district of Taquaral de Minas, Brazil, expressed as mean ± standard deviation. Asterisks (*) indicate significant differences (*p* < 0.05) for samples when compared to the negative control (C−). Abbreviations: BOD = Bode; PIR = Pirineu; PIN = Pinheira; LAJ = Lajedo; MAR = Marmita.

**Figure 10:**
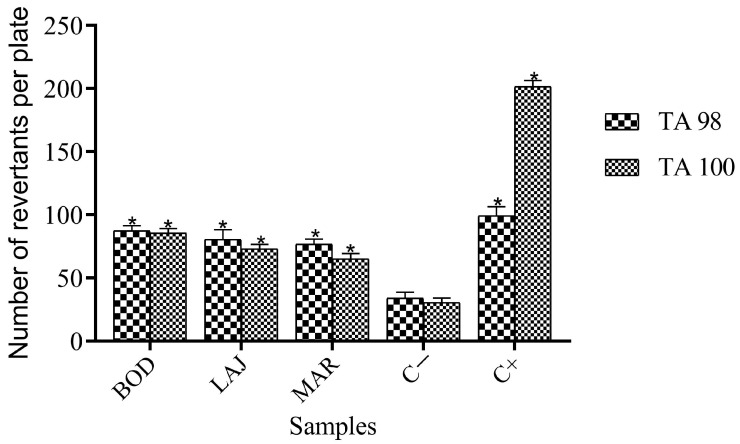
Results obtained for the Ames test with metabolization when testing aqueous extract of soil samples from mines in the district of Taquaral de Minas, Brazil, expressed as mean ± standard deviation. Asterisks (*) indicate significant differences (*p* < 0.05) for samples when compared to the negative control (C−). Abbreviations: BOD = Bode; PIR = Pirineu; PIN = Pinheira; LAJ = Lajedo; MAR = Marmita.

Micronutrients and mutagenic activities in the absence and presence of the S9 fraction were analyzed by calculating the MR from the statistical analyses (Graph Pad Prism 8.0.2 software), as shown in Table 5 and summarized in Figure 9 and Figure 10.

According to the results, it was found that all samples have an MR greater than or equal to 2 for both strains. Furthermore, statistical analyses confirmed the mutagenic potential of the samples under study, with a *p*-value < 0.05.

These results may be due to the presence of high levels of metals, as reported by Santos (2018) [17], because, in the inorganic fraction of the soil, toxic metals are a class of priority compounds in the definition of probable contaminants of anthropogenic or geological origin. These chemical elements can occur in natural environments and are essential to living beings, but in excessive amounts, they can be toxic or powerfully mutagenic, carcinogenic, and teratogenic agents [19,80].

Another factor that can justify the genotoxic effects in the samples is the presence of free radicals coming from silica and certain metals found in high concentrations [81]. The presence of these elements can destroy or react with practically all biological molecules, both in unicellular and multicellular organisms, inducing chromosomal aberrations, mutations, and exchanges of sister chromatids. In bacteria, the targets of radical species are lipids, proteins, DNA, and RNA. In DNA, reactive species act by causing lesions such as alterations or the removal of nitrogenous bases, causing mutations in the affected organisms [80,82].

Corroborating the results of the Ames test, some studies have investigated the mutagenic effects of some radical species on different strains of salmonella, including TA100 and TA98, using the pre-incubation method and board incorporation to determine the toxic potential of these substances [83,84].

## 4. Conclusions

In general, it can be stated that, for the enzymatic antioxidant catalase, there was an inhibition of its activity in all the examined groups (*p* < 0.05). Similarly, significant differences in the lipid peroxidation results were obtained when compared to the unexposed group (*p* < 0.05), demonstrating that individuals exposed to mining environments face greater oxidative stress. Furthermore, it has been demonstrated that oxidative effects may be associated with enhancing the development of cancer as a chronic occupational disease resulting from exposure to contaminants found in the mining environment. Despite the limitations of this study, such as the limited number of participants, the results provide a useful overview of the mechanisms of exposure in this mining environment.

Analyzing the micronutrients, it was found that the group of prospectors had lower mean copper and selenium concentrations than the negative controls. Thus, the decrease in mineral availability in these individuals negatively affected the activity of metalloenzymes such as catalase, important components in the antioxidant defense response.

The *Allium cepa* and Salmonella/microsome tests showed that the soil samples had cytotoxic and genotoxic potential; this was due to the presence of toxic compounds present in the soil. Although the number of micronuclei was not statistically significant in the mutagenicity test, numerically increased values were found, so prolonged exposure can be harmful to the exposed organisms, especially given the mutagenic potential of the samples revealed by the Ames test. Thus, the results of this study reinforce the need to include the Salmonella/microsome assay in health risk assessments related to exposure to contaminated soil.

## Figures and Tables

**Table 1:** Blood concentration (μg·L^−1^) of micronutrients for the control group and the exposed group; data presented as intervals and means.

Element	20–30	30–40	40–50	50–60	60–70	70–80	Control	R^2^
Fe	569,634.89	583,184.53	572,925.90	549,363.50 *	377,906.93	321,043.61 *	580,573.83	0.08
Cu	980.42	972.83 *	1154.78	937.96 *	488.55	747.94	1109.77	0.00
Zn	6798.32	6610.94	6832.31	6801.54	4454.86	4473.60	7213.15	0.13
Se	62.98	49.63 *	56.12 *	49.61 *	43.85 *	28.49 *	72.82	0.00

Source. Santos, 2018 [17]. Asterisks (*) indicate significant differences (*p* < 0.05) for samples when compared to the control (C). The determination coefficient (R^2^) refers to the correlation between micronutrients and CAT activity. The intervals 20–30, 30–40, 40–50, 50–60, 60–70, 70–80 refer to the subgroups of miners, categorized by age.

**Table 2:** Results of calculations of the mitotic index, average root growth, and inhibition rate observed upon analysis of 5000 meristematic cells of *Allium cepa* after exposure to aqueous extract of soil samples from mines in the Taquaral district of Minas Gerais, Brazil.

Samples	Number of Cells in Interphase	Number of Cells in Mitosis	Average Root Growth	Inhibition Rate (CTI) %	Mitotic Index(MI)
^1^C−	4493	507	2.04 ± 0.56	0	10.48 ± 2.48
^2^C+	4296	704	1.21 ± 0.57 *	40.53	14.10 ± 2.54 *
BOD P	4391	609	1.77 ± 0.71 *	18.42	12.18 ± 1.80
BOD PP	4393	607	1.66 ± 0.96 *	18.66	12.38 ± 2.02
BOD SS	4381	619	1.91 ± 0.77	6.30	12.14 ± 1.98
PIR P	4301	699 *	1.63 ± 0.71 *	20.06	13.98 ± 2.35 *
PIR PP	4309	691 *	1.54 ± 0.78 *	24.29	13.82 ± 2.38 *
PIR SS	4358	642	1.50 ± 0.70 *	26.33	12.84 ± 1.33
PIN P	4388	612	1.54 ± 0.59 *	24.14	12.24 ± 2.01
PIN PP	4305	695 *	2.18 ± 0.61	−7.12	13.90 ± 2.32 *
PIN SS	4402	598	2.16 ± 0.66	−6.08	11.96 ± 1.70
LAJ P	4384	616	2.11 ± 0.66	−3.74	12.32 ± 2.04
LAJ PP	4378	622	2.02 ± 0.67	1.04	12.44 ± 3.08
LAJ SS	4380	620	1.76 ± 0.48 *	13.61	12.40 ± 2.24
MAR P	4364	636 *	1.77 ± 0.81	12.90	12.72 ± 1.80 *
MAR PP	4322	678 *	2.13 ± 0.99	−4.52	13.56 ± 2.36 *
MAR SS	4397	603	1.44 ± 0.65 *	29.47	12.06 ± 1.18

1: Ultrapure water; 2: methyl methanesulfonate (MMS). The asterisks (*) indicate significant differences (*p* < 0.05) for samples when compared to the negative control (C−). Abbreviations: BOD = Bode; PIR = Pirineu; PIN = Pinheira; LAJ = Lajedo, MAR = Marmita; P = soil dust sample; PP = stone dust sample; and SS = soil sample.

**Table 3:** Results of chromosomal aberrations and micronuclei observed in 5000 meristematic cells of *Allium cepa* after exposure to aqueous extract of soil samples from mines in the district of Taquaral de Minas, Brazil, expressed as mean ± standard deviation.

Samples	Bridging	Loss	Breaking	Bridging and Loss	Bridging and Breaking	Loss and Breaking	C-Metaphase	Sticking	Total CA	Micronuclei
^1^C−	7 ± 0.48	1 ± 0.18	2 ± 0.36	0 ± 0.0	0 ± 0.0	0 ± 0.0	4 ± 0.64	0 ± 0.0	12 ± 3.48	4 ± 0.48
^2^C+	24 ± 2.08	32 ± 2.66	10 ± 1.60	4 ± 0.79	0 ± 0.0	1 ± 0.20	70 ± 2.60	21 ± 1.88	162 ± 35.56 *	31 ± 2.52 *
BOD P	8 ± 0.36	5 ± 0.36	3 ± 0.52	0 ± 0.0	0 ± 0.0	0 ± 0.0	20 ± 1.48	0 ± 0.0	36 ± 8.89 *	5 ± 0.50
BOD PP	17 ± 1.36	20 ± 0.42	7 ± 1.04	0 ± 0.0	0 ± 0.0	3 ± 0.0	54 ± 1.36	0 ± 0.0	101 ± 24.47 *	7 ± 0.56
BOD SS	10 ± 1.76	0 ± 0.0	1 ± 0.48	0 ± 0.0	0 ± 0.0	0 ± 0.0	22 ± 1.10	0 ± 0.0	33 ± 9.56	8 ± 0.48
PIR P	15 ± 1.9	21 ± 1.92	18 ± 2.52	0 ± 0.0	0 ± 0.0	0 ± 0.0	57 ± 2.90	8 ± 2.77	119 ± 27.36 *	10 ± 0.80
PIR PP	9 ± 0.90	0 ± 0.0	7 ± 0.84	0 ± 0.0	0 ± 0.0	0 ± 0.0	25 ± 1.40	5 ± 0.86	54 ± 12.22 *	4 ± 0.48
PIR SS	10 ± 0.70	10 ± 1.20	6 ± 0.98	0 ± 0.0	0 ± 0.0	0 ± 0.0	27 ± 0.0	5 ± 0.0	58 ± 13.16 *	3 ± 0.42
PIN P	15 ± 0.70	4 ± 0.64	4 ± 0.64	0 ± 0.0	0 ± 0.0	0 ± 0.0	27 ± 1.30	4 ± 0.72	54 ± 13.33 *	4 ± 0.48
PIN PP	11 ± 1.32	2 ± 0.32	0 ± 0.0	0 ± 0.0	0 ± 0.0	0 ± 0.0	21 ± 0.92	6 ± 0.96	40 ± 10.07 *	9 ± 0.54
PIN SS	17 ± 1.70	0 ± 0.0	0 ± 0.0	0 ± 0.0	6 ± 1.08	0 ± 0.0	27 ± 1.64	4 ± 0.72	57 ± 14.0 *	6 ± 0.7
LAJ P	19 ± 1.30	5 ± 0.96	7 ± 0.98	0 ± 0.0	0 ± 0.0	0 ± 0.0	45 ± 1.10	0 ± 0.0	76 ± 19.85 *	8 ± 0.48
LAJ PP	11 ± 0.90	11 ± 1.12	5 ± 0.70	0 ± 0.0	0 ± 0.0	0 ± 0.0	30 ± 1.68	12 ± 1.68	69 ± 15.18 *	9 ± 0.57
LAJ SS	23 ± 1.76	0 ± 0.0	3 ± 0.48	0 ± 0.0	0 ± 0.0	0 ± 0.0	20 ± 1.1	14 ± 1.48	60 ± 14.14 *	7 ± 0.4
MAR P	22 ± 2.0	7 ± 0.44	5 ± 0.70	0 ± 0.0	3 ± 0.54	0 ± 0.0	9 ± 1.08	0 ± 0.0	46 ± 10.56	4 ± 0.56
MAR PP	15 ± 1.4	13 ± 1.82	11 ± 1.12	0 ± 0.0	0 ± 0.0	0 ± 0.0	19 ± 1.50	6 ± 0.96	64 ± 12.30 *	7 ± 0.56
MAR SS	14 ± 1.68	9 ± 0.90	8 ± 0.98	3 ± 0.59	0 ± 0.0	5 ± 0.90	31 ± 1.88	7 ± 0.54	77 ± 16.40 *	7 ± 0.42

1: Ultrapure water; 2: methyl methanesulfonate (MMS). The asterisks (*) indicate significant differences (*p* < 0.05) for samples when compared to the negative control (C−) using the Mann–Whitney test. Abbreviations: BOD = Bode; PIR = Pirineu; PIN = Pinheira; LAJ = Lajedo; MAR = Marmita; P = soil dust sample; PP = stone dust sample; SS = soil sample; and CA = chromosomal aberrations.

**Table 4:** Results of the ANOVA test and multiple comparison between the studied groups for the parameters of mean root growth and chromosomal aberrations.

Samples	Chromosomal Aberrations	*p* Value	Average Root Growth	*p* Value
BOD P vs. PIR P	Yes	<0.001	No	>0.999
BOD PP vs. PIR PP	Yes	0.049	No	>0.999
BOD PP vs. PIN PP	Yes	<0.001	No	0.240
BOD SS vs. MAR SS	Yes	0.023	No	0.193
PIR P vs. LAJ P	Yes	<0.001	No	0.125
PIR P vs. MAR P	Yes	<0.001	No	>0.999
PIR PP vs. PIN PP	Yes	<0.001	Yes	0.016
MAR PP vs. PIR PP	No	>0.999	Yes	0.028
MAR SS vs. PIN SS	No	0.806	Yes	0.005
PIR SS vs. PIN SS	No	>0.99	Yes	0.019
PIN P vs. LAJ P	No	0.148	Yes	0.013

Abbreviations: BOD = Bode; PIR = Pirineu; PIN = Pinheira; LAJ = Lajedo; MAR = Marmita; P = soil dust sample; PP = stone dust sample; and SS = soil sample.

**Table 5:** Results obtained from the Ames test with metabolization when testing aqueous extract of soil samples from mines in the district of Taquaral de Minas, Brazil, expressed as mean ± standard deviation.

Samples	No Metabolization				With Metabolization			
	TA100 ^a^	MR	TA 98 ^b^	MR	TA 100 ^c^	MR	TA98 ^d^	MR
BOD	61 ± 2.0	3.21	69 ± 2.7	3.83	85.5 ± 2.9	2.80	88 ± 2.5	2.51
PIR	65.5 ± 4.5	3.45	38 ± 4.0	2.11	-	-	-	-
PIN	64 ± 2.0	3.37	40 ± 2.0	2.22	-	-	-	-
LAJ	71 ± 3.0	3.74	69 ± 3.6	3.83	72 ± 5.5	2.36	80.05 ± 2.7	2.29
MAR	76 ± 4.0	4.00	39.5 ± 0.5	2.19	65 ± 3.1	2.13	78 ± 3.0	2.23
C−	19 ± 3.0	-	18 ± 2.0	-	30.5 ± 3.3	-	35 ± 2.5	-
C+	84 ± 5.1	-	71 ± 3.3	-	201.5 ± 5.1	-	98 ± 3.5	-

Ultrapure water was used for items a, b, c, and d as C−. As C+, a = nitroquinoline, b = sodium azide, and c and d = aminoanthracene were used. Abbreviations: BOD = Bode; PIR = Pirineu; PIN = Pinheira; LAJ = Lajedo; MAR = Marmita.

## Data Availability

The datasets generated during the current study are available from the authors upon reasonable request.
